# Off-Grid Sparse Bayesian Learning for Channel Estimation and Localization in RIS-Assisted MIMO-OFDM Under NLoS

**DOI:** 10.3390/s25134140

**Published:** 2025-07-02

**Authors:** Ural Mutlu, Yasin Kabalci

**Affiliations:** 1 Bor Vocational School, Nigde Ömer Halisdemir University, Nigde 51700, Turkey; umutlu@ohu.edu.tr; 2 Department of Electrical and Electronics Engineering, Faculty of Engineering, Nigde Ömer Halisdemir University, Nigde 51240, Turkey

**Keywords:** reconfigurable intelligent surface, Off-Grid Sparse Bayesian Learning, compressed sensing: angle estimation, Orthogonal Matching Pursuit

## Abstract

Reconfigurable Intelligent Surfaces (RISs) are among the key technologies envisaged for sixth-generation (6G) multiple-input multiple-output (MIMO)–orthogonal frequency division multiplexing (OFDM) wireless systems. However, their passive nature and the frequent absence of a line-of-sight (LoS) path in dense urban environments make uplink channel estimation and localization challenging tasks. Therefore, to achieve channel estimation and localization, this study models the RIS-mobile station (MS) channel as a double-sparse angular structure and proposes a hybrid channel parameter estimation framework for RIS-assisted MIMO-OFDM systems. In the hybrid framework, Simultaneous Orthogonal Matching Pursuit (SOMP) first estimates coarse angular supports. The coarse estimates are refined by a novel refinement stage employing a Variational Bayesian Expectation Maximization (VBEM)-based Off-Grid Sparse Bayesian Learning (OG-SBL) algorithm, which jointly updates azimuth and elevation offsets via Newton-style iterations. An Angle of Arrival (AoA)–Angle of Departure (AoD) matching algorithm is introduced to associate angular components, followed by a 3D localization procedure based on non-LoS (NLoS) multipath geometry. Simulation results show that the proposed framework achieves high angular resolution; high localization accuracy, with 97% of the results within 0.01 m; and a channel estimation error of 0.0046% at 40 dB signal-to-noise ratio (SNR).

## 1. Introduction

Reconfigurable Intelligent Surfaces (RISs) have emerged as a new paradigm and a promising technology for enhancing wireless communication systems. Architecturally, an RIS is composed of a large array of passive reflecting elements that can independently manipulate the phase and amplitude of incoming signals, thereby improving spatial resolution and spectral efficiency in complex propagation environments [1,2]. In the sixth-generation (6G) wireless networks, RIS is expected to become one of the key enablers, not only for wireless communication, but also for sensing applications such as localization and environment mapping [3,4]. When integrated with massive multiple-input and multiple-output orthogonal frequency division multiplexing (MIMO-OFDM) and millimetre-wave (mmWave) communications, RIS-assisted systems can achieve increased spatial diversity and finer angular resolution to support high-precision localization [5,6,7].

In contemporary wireless communication systems, localization algorithms can be classified in two main categories: geometry-driven estimation, based on multipath parameters such as Angle of Arrival (AoA), Angle of Departure (AoD), and Time of Arrival (ToA) [8] and data-driven localization, based on deep learning and having previous knowledge of the environment [9]. The geometry-driven localization approach requires the estimation of channel or multipath parameters through which localization computations can be carried out. In traditional MIMO-mmWave communication systems with dominant line-of-sight (LoS) transmission path scenarios, localization is relatively easy to estimate, as the AoA–AoD and ToA parameters satisfy the conditions needed for estimating a directional vector and its distance. However, in dense urban environments, LoS paths are frequently blocked, rendering traditional localization strategies based on LoS ineffective, particularly in mmWave frequencies where the channels are often dominated by non-LoS (NLoS) multipath components.

In the dense urban environment scenario, RISs have the potential to provide controllable NLoS or Virtual LoS (VLoS) paths that facilitate localization where conventional methods fail [10,11,12]. RIS systems can further improve localization by providing an alternative path to the MS and additional location reference points. Nevertheless, in dense urban environments, even the RIS-MS VLoS path may be blocked, making it necessary to rely on NLoS multipath components. In the NLoS scenarios, the accuracy of channel estimation and localization depends on precise estimation of the AoA and AoD of the dominant multipath components. At mmWave frequencies, the propagation environment is typically characterized by angular sparsity due to high propagation loss and limited scattering [5,13]. This inherent sparsity forms the basis for many high-resolution channel estimation and localization techniques in mmWave systems. However, channel estimation in RIS-assisted systems has its distinct challenges [8,14,15,16]. First, the propagation path is divided into base station (BS)–RIS and RIS–mobile station (MS) channels, each requiring independent modelling. Second, RIS elements are passive and not capable of transmitting pilots, complicating parameter estimation. Third, the 2D structure of RIS, while enabling 3D beamforming, significantly increases the algorithmic complexity due to the enlarged angular search space.

Given these observations, there remains a significant gap in RIS-assisted localization under practical uplink NLoS scenarios, particularly for systems relying on passive RIS elements and compressed angular measurements. Most existing studies simplify the problem by assuming downlink estimation, LoS paths, or active RIS architectures. This work addresses this limitation by proposing a novel hybrid angular estimation and localization method for RIS-assisted MIMO-OFDM systems operating under realistic NLoS uplink conditions and utilizing the compressive structure of RIS.

### 1.1. Contributions of the Work

The main contributions of this work are as follows:

- The study models the RIS-MS channel as a block-sparse structure, where the RIS-side AoA induces row sparsity and the MS-side AoD induces column sparsity, with the RIS acting as a compressive sensing matrix. Assuming a known BS-RIS channel, this model enables uplink estimation of geometric parameters for NLoS paths.
- Hybrid Estimation Framework: A hybrid algorithm combining Simultaneous Orthogonal Matching Pursuit (SOMP) and Variational Bayesian Expectation Maximization (VBEM)-based Off-Grid Sparse Bayesian Learning (VB-OG-SBL) is proposed. SOMP provides coarse support estimation using a discrete dictionary, while VB-OG-SBL refines angular estimates. The hybrid protocol individually addresses the RIS-side AoA and MS-side AoD estimations.
- Off-Grid Angular Refinement via VB-OG-SBL: To address the off-grid angular problem, a VBEM-based OG-SBL framework is developed. It jointly refines azimuth and elevation offsets through second-order Newton-style updates within VBEM iterations, enabling stable and high-resolution continuous domain estimation.
- AoA–AoD Matching Strategy: A low-complexity matching algorithm is employed to associate RIS-side AoA and MS-side AoD estimates, resolving the decoupling ambiguity and enabling consistent path identification.
- 3D Localization from NLoS Paths: A geometric 3D localization algorithm is implemented to estimate the MS position by computing the orthogonal intersections of estimated NLoS paths. Based on MS localization, the scatterer positions are also inferred, enabling passive environmental sensing without requiring LoS paths.
- Performance Evaluation: Extensive simulations confirm that the proposed method achieves high angular accuracy, low channel reconstruction normalized mean square error (NMSE), and centimetre-level localization across a range of SNRs and multipath conditions.

### 1.2. Notations and Paper Outline

The rest of the paper is organized as follows. Section 2 presents the reference system architecture and introduces the sparse channel model. Section 3 describes the implementation of SOMP in the double sparsity structure. Section 4 describes the proposed VB-OG-SBL algorithm. Section 5 introduces the AoA–AoD matching protocol and 3D localization. Section 6 and Section 7 provide simulation results and concluding remarks, respectively. The notations used are as follows. Matrices are given with capital letters, e.g., A, while vectors are in bold lowercase letters, v. Transpose and Hermitian matrix operations are denoted as ${\left(.\right)}^{T}$ and ${\left(.\right)}^{H}$, respectively, while ${\left\|.\right\|}_{2}$ and ${\left\|.\right\|}_{F}$ represent the Euclidean and the Frobenius norms, respectively.

## 2. Related Work

This section reviews relevant work in two core domains: localization with RIS and sparse signal recovery techniques used for geometric channel estimation. First, we examine key studies in RIS-assisted positioning under LoS and NLoS scenarios. Then, we present foundational and recent developments in compressed sensing and sparse Bayesian methods that enable high-resolution angle estimation in RIS-based systems.

### 2.1. RIS-Assisted Localization

Due to the ability of RIS to introduce additional controllable multipath components, RIS-assisted localization studies are largely developed under the assumption that the direct path between the BS and MS is blocked and localization is performed through a VLoS path created by the RIS [10,11,17,18]. For example, earlier studies of [19,20] focus on single-input single-output (SISO) systems with a LoS between RIS and MS, estimating AoA–AoD and ToA for single-path localization, while the same model has also been extended to MIMO communications [21]. To improve angular diversity and robustness, multi-RIS-based localization strategies have also been proposed [18,19,21,22], offering additional geometric anchors that improve localization even in the absence of LoS. In one such study, ref. [10] models a multi-RIS architecture and derives the MS location based on the minimum distance between estimated angular direction vectors.

A major challenge arises in uplink channel estimation under passive RIS architectures. In such systems, the MS transmitted pilots traverse a cascaded path, MS-RIS-BS, resulting in a multiplicative channel structure involving RIS-MS and RIS-BS channels with a combined phase altered by the RIS reflection matrix. To solve some of the uplink problems, ref. [23] employs an SBL framework to estimate the angular-domain structure of the cascaded channel from MS to BS, and then, it is extrapolated across OFDM subcarriers, implicitly treating the RIS-MS link as embedded within it. Similarly, ref. [24] applies compressive sensing to estimate the end-to-end sparse vector without discretizing the RIS angular domain, thus bypassing explicit RIS-MS recovery. Furthermore, refs. [8,18] assume the RIS-BS channel properties are known and that RIS-MS can be extracted from the cascaded channel. Other methods involve equipping the RIS with active elements [16,25]. Active elements enable partial observation of incoming signals at the RIS, allowing independent estimation of the RIS-MS channel by enabling it to operate as a sensing node.

The literature on uplink-based channel estimation in passive RIS settings remains sparse, particularly under general NLoS conditions and uniform planar array (UPA) architectures. The reliance on downlink estimation limits real-time adaptability and optimization from the BS side. As such, accurate, uplink-based geometric channel estimation from the NLoS multipath remains a key challenge and a critical bottleneck for enabling practical RIS-assisted localization in 6G systems.

### 2.2. Sparse Signal Recovery (SSR)

Traditional SSR approaches include spectral subspace methods such as multiple signal classification (MUSIC) and estimation of signal parameters via the rotational invariance techniques (ESPRIT) [26,27], which offer high angular resolution but degrade under low SNR or under strongly correlated sources [28]. Greedy algorithms such as Matching Pursuit (MP), Compressive Sampling MP (CoSaMP), and Orthogonal MP (OMP) are better at handling coherent or highly correlated signals. OMP especially stands out due to its lower computational complexity, making it particularly attractive for real-time or resource-constrained systems [29,30]. Nonetheless, it can suffer from early selection errors and instability in low SNR or high compression [31]. Another major challenge in grid-based AoA estimation methods is the off-grid problem, where true source directions do not align with discretized angular grid points. The off-grid problem is usually addressed by either increasing grid resolution or by employing post-OMP refinement methods such as gradient descent, Taylor-series approximations, perturbation-based reweighting, etc. [32].

Sparse Bayesian Learning (SBL) is another SSR technique that has gained popularity in radar and array processing due to its robustness to noise and grid mismatch and its ability to automatically infer sparsity [33,34]. However, its higher computational complexity is a drawback, especially in RIS-assisted systems where both RIS and MS are implemented as UPAs, resulting in a quadratically increasing dictionary grid. This scalability issue is not limited to SBL only; any dictionary-based greedy algorithms also struggle to scale efficiently with large arrays or fine-resolution grids. To mitigate this, off-grid methods that are not strictly dependent on predefined dictionaries have been developed. Off-grid SBL (OG-SBL) is one such method designed to use a reduced dictionary to balance performance and complexity [35,36,37,38].

The number of studies that apply SBL-based AoA estimation in MIMO systems with UPAs is still limited, mainly due to the significantly increased dictionary size associated with 2D angle grids. To address this, several hybrid estimation approaches have been proposed. For instance, refs. [39,40] adopt a block-OMP method for elevation estimation followed by SBL for azimuth recovery using block-sparse priors to reduce complexity. Additional works such as [41] employ CoSaMP-SBL hybrid methods for joint azimuth–elevation estimation, where CoSaMP prunes the dictionary before Bayesian refinement. Off-grid SBL extensions in RIS-MIMO systems also exist: for instance, refs. [36,37] implement OG-SBL in UPA-based MIMO systems to refine AoA estimates. In [35], the concept is extended to active element-based RIS-assisted MIMO architectures to decouple the BS-RIS and RIS-MS channels, with Newton-based refinement of angular estimates. However, the estimates are largely based on the active elements. These strategies confirm that standard SBL alone is often impractical in high-dimensional UPA systems and that dimensionality reduction, hybridization, or structural sparsity must be taken into account for computational feasibility.

In RIS-assisted MIMO systems, acquiring full-dimensional array observations at the BS is not feasible due to excessive training overhead [33]. Therefore, a sampling matrix or a compressive sensing matrix is applied to reduce signal dimensionality prior to processing. In RIS-assisted systems, the reflection coefficients inherently act as a compression matrix, compressing spatial information [42,43]. Therefore, AoA estimation in RIS-assisted MIMO systems becomes a compressed sensing problem, where sparse angular support must be recovered from a subset of compressed measurements. Among the SSR methods, greedy algorithms like OMP and SBL adapt well to the compressive setting, while MUSIC and ESPRIT require significant adaptation [30,44].

### 2.3. Summary and Research Gap

The existing research on RIS-assisted localization largely focuses on downlink estimation strategies or assumes LoS availability, limiting their applicability in realistic NLoS environments. While multi-RIS and angular parameter-based methods have shown promise, they often neglect the uplink estimation bottleneck in passive RIS architectures. Hence, there remains a need for unified, low-complexity, and scalable uplink channel estimation and localization frameworks that operate effectively under passive RIS setups and NLoS propagation conditions, which constitutes the central focus of this study.

## 3. System Model

This section outlines the uplink transmission model for an RIS-assisted mmWave MIMO-OFDM system, emphasizing the geometric relationships and signal formulations necessary for sparse channel estimation.

### 3.1. System Reference Model 

**Figure 1:**
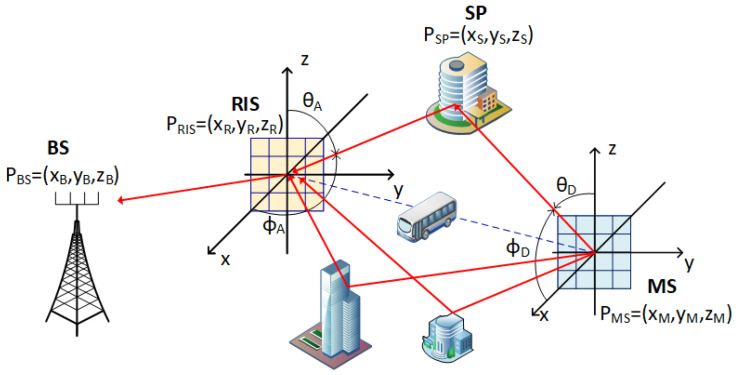
Reference model for urban RIS-assisted MIMO-OFDM communication system.

The reference model for the RIS-assisted mmWave MIMO-OFDM communication system is given in Figure 1. The communication system consists of a BS, RIS, and MS. Both the BS and the MS are equipped with UPA antennas of sizes $M=M_{x}\times M_{y}$ and $K=K_{x}\times K_{y}$, respectively. The reflecting elements of the RIS are also arranged in a UPA-like array of $L=L_{x}\times L_{y}$ elements. Communication occurs exclusively via the RIS, with no direct BS-MS link. This creates a cascaded channel composed of a BS-RIS sub-channel given with $H_{BR}\in {\mathbb{C}}^{M\times N}$ and the RIS-MS channel denoted as $H_{RM}\in {\mathbb{C}}^{N\times K}$. The RIS reflection pattern matrix is $\Psi =diag(e^{j\psi_{1}},e^{j\psi_{2}},\ldots ,e^{j\psi_{L}})\in {\mathbb{C}}^{L\times L}$, where $\psi_{l}\in [0,\;2\pi )$ is the reflection phase of the *l*th reflection element. The main assumption of the study is that the communication system is deployed in a dense urban environment scenario where the LoS path between the RIS and the MS may often become obstructed and the RIS-MS channel consists mainly of NLoS multipath components. In addition, the BS and the RIS positions and orientations are known, making $H_{BR}$ and its geometric parameters of AoA, AoD, and ToA known. OFDM has N subcarriers, bandwidth B, and subcarrier spacing $f_{s}=B/N$.

Figure 1 also shows the coordinate system adopted. A wave propagation direction is specified by an azimuth angle $\phi \in [0^{{}^{\circ}},360^{{}^{\circ}})$ measured from the positive x-axis and an elevation angle $\theta \in [-90^{{}^{\circ}},90^{{}^{\circ}}]$ measured from the z-axis. The 3D positions of the communicating nodes are given as $P_{B}={\left[x_{B},y_{B},z_{B}\right]}^{T}\in {\mathbb{R}}^{3}$, $P_{R}={\left[x_{R},y_{R},z_{R}\right]}^{T}$, and $P_{M}={\left[x_{M},y_{M},z_{M}\right]}^{T}$ for the BS, RIS, and MS, respectively, while the locations of the single bounce scatter points are in the $P_{S}^{\left(j\right)}={\left[x_{S},y_{S},z_{S}\right]}^{T}$ format, where j denotes the *j*th multipath.

### 3.2. Channel Model

In the mmWave frequency spectrum, high path loss and limited scattering lead to angular sparsity and a small number of dominant multipath components [5]. This results in a situation where each multipath component is defined by its AoA, AoD, ToA, and path gain. Therefore, the channel can be represented as a superposition of steering vectors weighted by these path gains. For UPAs, the steering vector is formed as a Kronecker product of horizontal and vertical components, denoted by $a_{x}$ and $a_{y}$, respectively. The full expressions are given in Equations (1)–(3).

(1) $$a_{x}(\phi ,\theta )={\left[1,e^{j2\pi \frac{d_{x}}{\lambda }\cos(\phi )\sin(\theta )},\ldots ,e^{j2\pi \frac{d_{x}}{\lambda }(N_{x}-1)\cos(\phi )\sin(\theta )}\right]}^{T}$$

(2) $$a_{y}(\phi ,\theta )={\left[1,e^{j2\pi \frac{d_{y}}{\lambda }\sin(\phi )\sin(\theta )},\ldots ,e^{j2\pi \frac{d_{y}}{\lambda }(N_{y}-1)\sin(\phi )\sin(\theta )}\right]}^{T}$$

(3) $$a(\phi ,\theta )=a_{x}(\phi ,\theta )⊗a_{y}{\left(\phi ,\theta \right)}^{T}$$

In the equations, $d_{x}$ and $d_{y}$ are the horizontal and vertical distances between the adjacent array elements. Using the steering vector equations, the BS-RIS channel for the *n*th subcarrier with a single LoS component is given as follows [45]:

(4) $$H_{BR,n}=\chi e^{-j2\pi \tau_{BR}\frac{(n-1)B}{N}}a_{B}(\phi_{B},\theta_{B})a_{R}{\left(\phi_{R},\theta_{R}\right)}^{H}$$

where $\tau_{BR}$ is the channel time delay, χ is the path gain, and $\left(\phi_{B},\theta_{B}\right)$ and $\left(\phi_{R},\theta_{R}\right)$ are the AoA and AoD angle pairs at the BS and the RIS, respectively. The RIS-MS channel, on the other hand, comprises J multipath components, and the channel is modelled as the sum of J steering vectors:

(5) $$H_{RM,n}=\sum_{j=1}^{J}\rho_{j}e^{-j2\pi \tau_{RM,j}\frac{(n-1)B}{N}}a_{R}(\phi_{R,j},\theta_{R,j})a_{M}{\left(\phi_{M,j}\theta_{M,j}\right)}^{H}$$

In the equation, $\rho_{j}$ is the path gain, $\tau_{RU,j}$ is the path delay, while the $\left(\phi_{R,j},\theta_{R,j}\right)$ and $\left(\phi_{M,j}\theta_{M,j}\right)$ pairs indicate the AoA at the RIS and AoD at the MS for the *j*th multipath. Location-dependent geometric parameters of angle and delay are calculated as follows:

(6) $$\theta_{R,j}=\arcsin(\left(P_{S}^{z,(j)}-P_{B}^{z}\right)/{\left\|P_{S}^{\left(j\right)}-P_{B}\right\|}_{2})$$

(7) $$\phi_{R,j}=\arctan(\left(P_{S}^{y,(j)}-P_{B}^{y}\right)/\left(P_{S}^{x,(j)}-P_{B}^{x}\right))$$

(8) $$\phi_{M,j}=\arctan(\left(P_{M}^{y}-P_{S}^{y,(j)}\right)/\left(P_{M}^{x}-P_{S}^{x,(j)}\right))$$

(9) $$\theta_{M,j}=\arcsin(\left(P_{M}^{z}-P_{S}^{z,(j)}\right)/{\left\|P_{M}-P_{S}^{\left(j\right)}\right\|}_{2})$$

(10) $$\tau_{RM,j}=\left({\left\|P_{S}^{\left(j\right)}-P_{B}\right\|}_{2}+{\left\|P_{M}-P_{S}^{\left(j\right)}\right\|}_{2}\right)/c$$

In the equations, *c* is the speed of light. Also, it should be noted that BS-RIS parameters are ignored.

### 3.3. Sparse Model of the Received Signal

Let the MS transmit a pilot sequence $x_{n}\in {\mathbb{C}}^{K\times 1}$ in the uplink direction; the received pilot signal $y_{n}\in {\mathbb{C}}^{M\times 1}$ at the BS for the *n*th subcarrier over a single OFDM symbol is

(11) $$y_{n}=H_{BR,n}\Psi H_{RM,n}x_{n}+z_{n}$$

In the equation, $z\sim \mathbb{N}(0,\sigma^{2}1_{M})$ is the Additive White Gaussian Noise (AWGN) added at the receiver.

In order to apply compressive sensing-based algorithms, the channel in (11) should be represented in terms of dictionary matrices and the sparsity matrices [5]. Hence, $H_{BR}$ is a LoS channel with known sparsity; it is not presented in a sparse form. The sparse representation of the RIS-MS channel in the angular domain is as follows:

(12) $$H_{RM}=A_{R}{ϒ}_{RM}A_{M}^{H}$$

In the equations, $A_{R}=[a_{R}(\phi_{1},\theta_{1}),\ldots ,a_{R}(\phi_{G_{R}},\theta_{G_{R}})]\in C^{L,{G_{R}}^{2}}$ and $A_{M}=[a_{M}(\phi_{1},\theta_{1}),\ldots ,a_{M}(\phi_{G_{M}},\theta_{G_{M}})]\in C^{K,{G_{M}}^{2}}$ are overcomplete dictionary matrices of array steering vectors at the RIS and MS nodes with resolutions of ${G_{R}}^{2}$ and ${G_{M}}^{2}$, respectively. The columns of the dictionary matrices correspond to array steering vectors of a specific azimuth and elevation angle pair in the AoA or AoD, and the resolutions of ${G_{R}}^{2}$ and ${G_{M}}^{2}$ are obtained based on equal horizontal and vertical resolutions. ${ϒ}_{RM}\in C^{{G_{R}}^{2}\times {G_{M}}^{2}}$ is a row–column–block sparsity matrix with only a few non-zero elements that select specific elements from the dictionary matrices. In the block sparsity, the row sparsity corresponds to the AoA dictionary atoms at the RIS side, and the column sparsity corresponds to the AoD dictionary atoms at the MS side. Rewriting (11) leads to

(13) $$y_{n}=H_{BR}\Psi A_{R}{ϒ}_{RM}A_{M}^{H}x_{n}+z_{n}$$

In the equation, $H_{BR}$ is not in a sparse format and can be reduced to its sub-elements to make the equation simpler. Taking into account that $H_{BR}$ consists of a BS and an RIS steering vector, the BS steering vector can be projected out by performing element-wise multiplication of the received signal $y_{n}$ with the known vector $a_{B}{\left(\phi_{B},\theta_{B}\right)}^{*}$. On the other hand, the BS-RIS side RIS steering vectors’ spatial frequencies can be subtracted from the RIS-MS side RIS steering vector. The simplified equation is given below:

(14) $${\hat{y}}_{n}=1_{M}\psi^{T}{\hat{A}}_{R}{ϒ}_{RM}A_{M}^{H}x_{n}+{\hat{z}}_{n}$$

In the equation, $1_{M}$ is a vector of 1 s denoting that the received signal for all the BS antennas is the same but with an i.i.d. noise. $\psi ={\left[\psi_{1},\psi_{2},\ldots ,\psi_{L}\right]}^{T}$ is the vector representation of the RIS coefficients, and ${\hat{A}}_{R}$ is obtained by subtracting the RIS angles. Also, ${\hat{y}}_{n}=y_{n}⊙a_{B}{\left(\phi_{B},\theta_{B}\right)}^{*}$ and ${\hat{z}}_{n}=z_{n}⊙a_{B}{\left(\phi_{B},\theta_{B}\right)}^{*}$ are the received signal and the noise.

## 4. Coarse Channel Estimation

**Figure 2:**
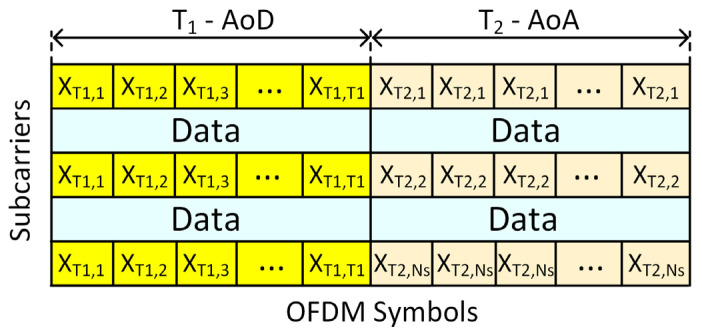
Two-phase channel parameter estimation.

Equation (14), obtained in the previous section, is in a block-sparsity structure format, where the row sparsity component represents AoA sparsity at the RIS and column sparsity represents AoD sparsity at the MS. Therefore, a two-phase coarse channel parameter estimation protocol based on double sparsity is proposed. The first phase estimates coarse AoDs at the MS, and the second phase estimates coarse AoAs at the RIS. Compared with standard double-sparsity structures, the MS side of the equation appears to be in a directly usable format [5,7,46]; thus, AoDs at the MS can be estimated without any further modifications. On the other hand, the RIS side of the equation requires further modifications. The two-phase protocol and the distribution of the pilot sequences in the OFDM grid are depicted in Figure 2.

### 4.1. Phase 1: AoD Estimation at MS

In Figure 2, the AoD estimation phase is denoted as T_1_ and consists of T_1_ OFDM symbols. For each symbol, a pilot sequence $x_{t}\in C^{K\times 1}$ from an orthogonal pilot matrix $X_{p}=(x_{1},x_{2},\ldots ,x_{T_{1}})\in {\mathbb{C}}^{K\times K}$ is transmitted from the MS. At the BS, T_1_-received pilot signals are concatenated to form the received signal, $Y_{MS,n}={\left({\hat{y}}_{1,n},{\hat{y}}_{2,n},\ldots ,{\hat{y}}_{T_{1},n}\right)}^{T}\in {\mathbb{C}}^{M\times T_{1}}$. By applying Hermitian operation to the received signal, the received signal becomes

(15) $$Y_{MS,n}^{H}=X_{p}^{H}A_{M}{ϒ}_{RM}^{H}{\hat{A}}_{R}^{H}\psi^{*}1_{M}^{T}+Z^{H}$$

where $Z\in {\mathbb{C}}^{M\times T_{1}}$ is the concatenated noise matrix.

As already indicated by the size of the pilot matrix, the training duration is equal to the MS array size, $T_{1}=K$, allowing the use of orthogonal training sequences of length K. The use of a full orthogonal matrix is feasible due to the limited dimensionality of the UPA at the MS (e.g., 4 × 4). By employing orthogonal pilot signals, the BS can eliminate the pilot from the received signal through correlation, resulting in the dictionary matrix becoming the effective sensing matrix. In order to perform SSR, RIS phase coefficients are assigned random complex values of the form $e^{j\upsilon }$, where $\nu \in \left[0,2\pi \right)$ is kept constant during the training period or over T_1_ symbols. This results in a constant $\zeta ={\hat{A}}_{R}^{H}\psi^{*}1_{M}^{T}$ that does not affect the sparsity.

After applying the orthogonal pilot matrix $X_{p}$ to the Hermiton of the received signal $Y_{MS,n}^{H}$, Equation (15) is transformed into a standard row-sparse representation suitable for SOMP:

(16) $${\hat{Y}}_{MS,n}=A_{M}{ϒ}_{RM}\zeta +\hat{Z}$$

In the equation, ${\hat{Y}}_{MS,n}^{H}\in {\mathbb{C}}^{T_{1}\times M}$ corresponds to M observations over T_1_ OFDM symbols and a single OFDM subcarrier. These M observations correspond to a rank-1 multiple measurement vector (MMV) model, where all measurement vectors are linear combinations of a single sparse vector and differ only in noise. Hence, ToA estimation requires multiple observations over multiple OFDM subcarriers, and the training signal in (11) is also applied to multiple OFDM subcarriers. In OFDM, each subcarrier has a slightly different phase shift due to frequency-dependent delay, which introduces subtle but critical frequency diversity to the observations [47,48]. To recover the coarse AoD support estimates from (16), the SOMP algorithm is employed. The SOMP algorithm processes multiple observations simultaneously under a common sparsity constraint. The algorithm is outlined in Algorithm 1.
**Algorithm 1:** SOMP algorithm for AoD estimation at MS 1: **Input:**
$Y_{MS}$
$,\;A_{M}$
2: **Parameter setting:**
 $R=Y_{MS}$
, Ω=∅ initialize residual and index set 
3: **for**
j≤J
**do**

4: $\;\;\;\;\;\;i=\arg\max{\left\|A_{M}^{H}R\right\|}_{2}^{2}$; calculate max values, estimate index of max values
5: $\;\;\;\;\;\;\Omega =\Omega \cup \left\{i\right\}$; update indices set 
6: $\;\;\;\;\;\;R=(I-A_{M}(\Omega ).A_{M}^{†}(\Omega ))Y_{MS}$; update residual 
7: **end for**

8: **Output:** Ω, from index set Ω get AoDs, max values

### 4.2. Phase 2: AoA Estimation at RIS

Phase 2 is shown as T_2_ in Figure 2 and consists of T_2_ OFDM symbols. It was shown in Equation (14) that the received signal at the BS is the same for all BS antennas but with different i.i.d. noise. Therefore, to carry out AoA estimation, the estimation protocol needs to be extended in the time domain as opposed to the spatial domain. Let $\Phi ={\left[\psi_{1},\psi_{2},\ldots ,\psi_{T_{2}}\right]}^{T}\in {\mathbb{C}}^{T_{2}\times L}$ be the RIS pattern applied to (14) for a T_2_ OFDM symbol; the received signal observed on a per-BS-antenna basis is as follows:

(17) $${\tilde{y}}_{n}=\Phi {\hat{A}}_{R}{ϒ}_{RM}\xi +{\tilde{z}}_{n}$$

In the equation, assuming $\xi =A_{M}^{H}x_{n}$ is constant over T_2_ symbols, $\tilde{y}\in {\mathbb{C}}^{T_{2}\times 1}$ is in row-sparsity format with the RIS reflection coefficients effectively becoming a compression matrix. Similar to the AoD estimation phase, multiple BS antennas produce multiple observations that are effectively rank-1 MMVs. AoA estimation observations are obtained in a manner similar to AoD, i.e., a set of OFDM subcarriers is used to produce MMVs. The SOMP algorithm for the AoA estimation at the RIS is the same as Algorithm 1, albeit with a different sensing matrix. The sensing matrix in Phase 2 is $\Theta =\Phi A_{R}$. In this phase, both the RIS coefficients and the $x_{n}$ pilot sequences are drawn from a Bernoulli distribution, taking the values of ±1 with the objective of approximating Restricted Isometry Property (RIP) [46,49].

## 5. Off-Grid SBL Refinement

In this section, we develop a variational Bayesian framework for refining the angular parameters associated with each source. The refinement process jointly updates the azimuth and elevation deviations within each VBEM iteration, enabling high-resolution off-grid estimation. Unlike MAP or standard EM methods, the VBEM algorithm leverages a mean-field variational approximation, which allows closed-form updates of the posterior and avoids overfitting by marginalizing latent variables rather than point-estimating them [50]. By integrating Newton-based off-grid angular refinement directly into the iterative updates, the framework reduces basis mismatch without relying on a fixed discrete grid. The proposed algorithm is explained through AoA estimation at the RIS side, which can be adopted to the MS side AoD estimation by simply assuming the compression matrix is an identity matrix. In the following section, for simplicity, $A_{R}={\hat{A}}_{R}$ and $y=\tilde{y}$.

### 5.1. Off-Grid Formulation

Equations (16) and (17) are the sparsity equations that may result in energy leakage due to the true angles being in between dictionary atoms. If Equations (16) and (17) are rewritten as functions of continuous angles, the received signal can be shown as follows:

(18) $$Y_{R}=\Phi A_{R}(\phi_{R},\theta_{R})S+Z$$

where the steering matrix $A_{R}(\phi_{R},\theta_{R})=[a_{R,1},\ldots ,a_{R,J}]\in {\mathbb{C}}^{L\times J}$ is parameterized by continuous azimuth and elevation angles as opposed to discrete angles in the SOMP method. In addition, $S\in {\mathbb{C}}^{J\times N_{Obs}}$ contains the channel gains of the *N_Obs_* observations. The objective of the proposed algorithm is to estimate the angular parameters ${\left(\phi_{R,j},\theta_{R,j}\right)}_{l=j}^{J}$, given the observations $Y_{R}$, as well as the sparse source structure S under the assumption that only J directions are active.

### 5.2. Sparse Bayesian Prior Structure

In order to promote sparsity in the source signals and enable automatic relevance determination (ARD), the study adopts a two-layer sparse Bayesian modelling framework [51,52]. In the first layer, each row $s_{j}\in {\mathbb{C}}^{1\times N_{Obs}}$ of the source matrix $S\in {\mathbb{C}}^{J\times N_{Obs}}$ represents the time-varying coefficients corresponding to a hypothesized source direction $\left(\phi_{j},\theta_{j}\right)$. We place independent zero-mean complex Gaussian priors over each row:

(19) $$p(s_{j}|\alpha_{j})=\prod_{k=1}^{N_{Obs}}CN(s_{j,n}|0,\alpha_{j}^{-1})=CN(0,\alpha_{j}^{-1}I_{J})$$

where $\alpha_{j}$ is a precision (inverse variance) parameter associated with the *j*th direction and k is the observation index. Large values of $\alpha_{j}$ result in the corresponding $s_{j}$ going towards zero, effectively making it sparse. In the second layer, each $\alpha_{j}$ follows an independent Gamma distribution:

(20) $$p(\alpha_{j})=\mathrm{Gamma}(\alpha_{j}|a,b)=\frac{b^{a}}{\Gamma (a)}\alpha_{j}^{a-1}e^{-b\alpha_{j}}$$

where a>0 and b>0 are hyperparameters, typically chosen to be small to enforce sparsity-promoting behaviour. The noise in the observations is modelled as additive complex Gaussian noise with precision $\beta =1/\sigma^{2}$:

(21) $$p(y_{k}|S,\beta )=CN(\Phi A(\phi ,\theta )s_{k},\beta^{-1}I_{J})$$

(22) $$p(Z)=\prod_{k=1}^{N_{Obs}}CN(y_{k}|\Phi A(\phi ,\theta )s_{k},\beta^{-1}I_{J})$$

In practice, β may be estimated jointly with the other parameters or fixed based on prior knowledge. The joint distribution over all unknown variables is then

(23) $$p(Y_{R},S,\alpha )=\prod_{k=1}^{N_{Obs}}p(y_{k}|S,\beta )\prod_{j=1}^{J}p(s_{j}|\alpha_{j})p(\alpha_{j})$$

The resulting probabilistic model defines a joint distribution over the observations *Y*, the latent source signals *S*, and the hyperparameters $\alpha =[\alpha_{1},\ldots ,\alpha_{J}]$.

### 5.3. Variational Inference Framework

Direct computation of the posterior $p(S,\alpha |Y_{R})$ is intractable due to the coupling between the latent variables in both the likelihood and the hierarchical prior structure. To address this, we employ a VBEM framework, where the joint posterior is approximated via a mean-field factorization as follows [51,53]:

(24) q(S,α)=q(S)q(α)

where posterior independence between the signal coefficients S and the precision parameters α is assumed, enabling iterative updates by optimizing each variational factor while keeping the other fixed. The variational inference procedure minimizes the Kullback–Leibler (KL) divergence between the true and approximate posteriors, which is equivalent to maximizing the evidence lower bound (ELBO):

(25) $$L(q)=E_{q}[\log p(Y,S,a)]-E_{q}[\log q(S)]-E_{q}[\log q(a)]$$

Due to the conjugacy between the Gaussian prior on *S* and the Gamma prior over α, both posterior distributions retain closed-form variational updates. Specifically, the variational distribution q(S) becomes a multivariate complex Gaussian, while $q(\alpha_{j})$ remains a product of Gamma distributions. Given the factorization in (25), VBEM iteratively updates these posteriors using closed-form E- and M-steps followed by an off-grid angular refinement step. The full VBEM procedure is outlined in Algorithm 2.

The first step of the VB-OG-SLB algorithm is the initialization stage, during which the angle refinements are set to zero and the model hyperparameters are initialized. Specifically, the hyperparameters governing the prior distributions are initialized using the signal power estimates obtained from the SOMP stage. The shape parameter a is set to the number of multipath components, while the scale parameter b is set to the normalized signal power associated with each path. This initialization ensures that the algorithm starts from a physically meaningful prior and promotes faster and more stable convergence in VBEM iterations.
**Algorithm 2:** VB-OG-SBL for AoA estimation at RIS **Input:** $Y_{R}$$,\;\Theta =\Phi A_{R}$$,\;\mathrm{coarse}\;(\phi_{j},\theta_{j})$J, signal powers **1. Initialize:** $\delta_{j}^{az}=0$$,\;\delta_{j}^{el}=0$, initialize hyperparameters
α, and noise precision
β **2. Repeat:** $\;\;\;\;\;\;2.1.\;\mathrm{Update}\;\mathrm{Steering}\;\mathrm{Vectors}:\;\mathrm{Recompute}\;\mathrm{with}\;\mathrm{updated}\;\mathrm{angles}\;A_{R}(\phi_{j}+\delta_{j}^{az},\theta_{j}+\delta_{j}^{el})$ $\;\;\;\;\;\;2.2.\;E-\mathrm{Step}:\;\mathrm{Compute}\;\mathrm{posterior}\;\mathrm{mean}\;\mu_{j}$$\;\mathrm{and}\;\mathrm{covariance}\;\Sigma_{j,j}$ of S
      2.3. M−Step: Update hyperparameters α and noise precision β
$\;\;\;\;\;\;2.4.\;\mathrm{Update}\;\mathrm{Angles}:\;\mathrm{Apply}\;\mathrm{Newton}\;\mathrm{updates}\;\mathrm{to}\;\mathrm{refine}\;\delta_{j}^{az}$
$\;\mathrm{and}\;\delta_{j}^{el}$
$\;\;\;\;\;\;2.5.\;\mathrm{Check}\;\mathrm{Convergence}:\;\mathrm{stop}\;\mathrm{if}\;\left\|\delta_{j}^{\left(t\right)}-\delta_{j}^{\left(t-1\right)}\right\|<\varepsilon$ or max iteration reached
**3. Return:** Refined angles and signals posteriors 
**Output:**$\;\mathrm{Refined}\;\mathrm{angles}\;\phi_{j}+\delta_{j}^{az}$
$,\;\;\theta_{j}+\delta_{j}^{el}$
$,\;\mathrm{posterior}\;\mathrm{means}\;\mu_{j}$ signal estimates
S


Following initialization, each VBEM iteration begins by updating the steering vectors using the most recent angular estimates. Let $A=A_{R}(\phi_{\left(i-1\right)},\theta_{\left(i-1\right)})\in C^{L\times J}$ denote the current steering matrix based on the latest angle estimates or iteration (i−1). Upon updating the vectors, the compressed dictionary becomes $\tilde{A}=\Phi A\in {\mathbb{C}}^{T_{2}\times J}$, which is the effective measurement matrix in the VBEM inference loop.

Given the current estimate of the dictionary $\tilde{A}$ and the precision vector $\alpha ={\left[\alpha_{1},\ldots ,\alpha_{J}\right]}^{T}$, the variational posterior over the sparse source matrix S is assumed to factorize across observations:

(26) $$q(S)=\prod_{k=1}^{N_{Obs}}CN(\mu_{k},\Sigma )$$

where $\mu_{k}\in {\mathbb{C}}^{J}$ is the posterior mean and $\Sigma \in {\mathbb{C}}^{J\times J}$ is the posterior covariance, which is identical across snapshots due to independence. The covariance is given by

(27) $$\Sigma ={\left(diag(\alpha )+\beta \;{\tilde{A}}^{H}\;\tilde{A}\right)}^{-1}$$

The posterior mean for each observation is given as

(28) $$\mu_{k}=\beta \Sigma \;{\tilde{A}}^{H}y_{k}$$

The posterior distribution over each precision parameter $\alpha_{j}$ remains Gamma distributed. To update the precision parameter, the expected signal energy is calculated as

(29) $$E\left[\left|s_{j}^{2}\right|\right]=\sum_{k=1}^{N_{Obs}}|\mu_{j,k}{|}^{2}+N_{Obs}\Sigma_{j,j}$$

where $\mu_{j,k}$ is the *j*th entry of the posterior mean and $\Sigma_{j,j}$ is the *j*th diagonal entry of the posterior covariance. The update function for the shape parameter and rate parameter of the Gamma distribution are

(30) $$a_{j}=a_{0}+N_{Obs}$$

(31) $$b_{l}=b_{0}+\frac{1}{2}E[|s_{j}{|}^{2}]$$

Thus, the expected precision becomes

(32) $$\alpha_{j}=\frac{a_{0}+N_{Obs}}{b_{0}+\frac{1}{2}E[|s_{j}{|}^{2}]}$$

The precision update balances the empirical signal energy at each index *j* with the associated uncertainty captured, leading to automatic sparsity pruning as irrelevant components are suppressed. It is this update of the $\alpha_{j}$ value that makes VBEM-based OG-SBL different than EM-based OG-SBL. If the noise variance $\sigma^{2}$ and the noise precision β are not known, then the noise precision is updated in closed form:

(33) $$\beta =\frac{J\times N_{Obs}}{\sum_{k=1}^{N_{Obs}}{\left\|y_{k}-\tilde{A}\mu_{k}\right\|}^{2}+N_{Obs}\times tr\left(\tilde{A}\Sigma {\tilde{A}}^{H}\right)}$$

The angular refinement step uses a second-order Newton update to adjust the azimuth and elevation angles of each active component [35,54,55]. Newton-style updates improve both accuracy and stability by using second-order derivatives to determine not only the direction but also the magnitude of each refinement step. Unlike first-order methods such as basic gradient descent, which can suffer from slow convergence or instability, Newton updates adapt to the local curvature of the error surface, resulting in more stable and efficient convergence. The Newton updates are derived by minimizing the expected data-fitting error under the current variational posterior. Specifically, the update approximates the gradient of the ELBO with respect to the angular deviations based on the current posterior mean estimates $\mu_{j,k}$ for each source j and observation k. This approach avoids explicitly computing the ELBO, while still enabling tractable second-order optimization.

Let the current azimuth and elevation angles for source *j* be represented as $\phi_{j}+\delta_{j}^{az}$ and $\;\;\theta_{j}+\delta_{j}^{el}$, where $\phi_{j}$ and $\;\;\theta_{j}$ are the coarse initial estimates, and $\delta_{j}^{az}$ and $\;\;\delta_{j}^{el}$ are the small refinement deviations to be learned. To update $\left(\phi_{j},\theta_{j}\right)$, we minimize the expected squared error for each observation k, defined as follows:

(34) $$E_{k}(\phi ,\theta )=E_{q(S)}\left[{\left\|y_{k}-\tilde{A}\mu_{k}\right\|}^{2}\right]$$

This cost function approximates the negative gradient of the ELBO with respect to the angular deviations. To refine the angular deviations, the expected squared fitting error contributed by the *j*th source component across all observations is expressed with the following cost function:

(35) $$L(\delta_{j}^{az}\;,\delta_{j}^{el}\;)=\sum_{k=1}^{N_{Obs}}\;\;{\left\|r_{k,j}\;-\mu_{j,k}{\tilde{\;a}}_{j}\;(\phi_{j}\;+\delta_{j}^{az}\;\;,\theta_{j\;}+\delta_{j}^{el}\;\;)\right\|}^{2}\;$$

where $r_{k,j}$ is a residual signal that isolates the contribution of the *j*th source:

(36) $$r_{k,j}=y_{k}-\sum_{g\neq j}{\tilde{a}}_{g}\mu_{g,k}$$

and where ${\tilde{a}}_{g}$ is the *g*th column of $\tilde{A}$ and $\mu_{g,k}$ is the posterior mean coefficient for source g and observation k.

Since the coarse angles are treated as constants during refinement, the gradients are computed with respect to the current total angles. Let $\frac{\partial {\tilde{a}}_{j}}{\partial \delta_{j}^{az}}$ and $\frac{\partial {\tilde{a}}_{j}}{\partial \delta_{j}^{el}}$ denote the derivatives of the steering vector with respect to azimuth and elevation deviations. The gradient of the expected cost with respect to $\delta_{j}^{az}$ and $\delta_{j}^{el}$ are

(37) ∂L∂δjaz=−2Re∑k=1NObsμj,k∗∂a˜jH∂δjazrk,jand∂L∂δjel=−2Re∑k=1NObsμj,k∗∂a˜jH∂δjelrk,j

To enable fast convergence, the study adopts a Newton-style update using approximate second-order information [35,56]. The Hessian terms are approximated by ignoring cross-terms and treating $\mu_{j,n}$ as constant over refinement steps:

(38) $$\frac{\partial^{2}L}{\partial \delta_{j}^{az2}}\approx 2\sum_{k=1}^{N_{Obs}}|\mu_{j,k}{|}^{2}{\left\|\frac{\partial {\tilde{a}}_{j}}{\partial \delta_{j}^{az}}\right\|}^{2}\mathrm{and}\;\frac{\partial^{2}L}{\partial \delta_{j}^{el2}}\approx 2\sum_{k=1}^{N_{Obs}}|\mu_{j,k}{|}^{2}{\left\|\frac{\partial {\tilde{a}}_{j}}{\partial \delta_{j}^{el}}\right\|}^{2}$$

The gradients in (38) approximate the direction of steepest descent of the ELBO with respect to the angle deviations, enabling fine-grained correction of the estimated azimuth and elevation values. Using these approximations, the Newton updates for the angular deviations are

(39) $$\delta_{j}^{az}\leftarrow \delta_{j}^{az}-\eta \frac{\partial L/\partial \delta_{j}^{az}}{\partial^{2}L/\partial \delta_{j}^{az2}}\;\mathrm{and}\;\delta_{j}^{el}\leftarrow \delta_{j}^{el}-\eta \frac{\partial L/\partial \delta_{j}^{el}}{\partial^{2}L/\partial \delta_{j}^{el2}}$$

where η∈(0,1] is an adaptive step size that decays over iterations.

The algorithm converges when the deviation in the estimated angles between two consecutive iterations fall below a predefined threshold or when the maximum number of iterations is reached. The convergence criteria is as follows:

(40) $$delta\_change=\frac{{\left\|\delta^{\left(i\right)}-\delta^{\left(i-1\right)}\right\|}_{2}}{{\left\|\delta^{\left(i-1\right)}\right\|}_{2}}\;\;\;<tolerance\_value$$

Although the ELBO objective could be monitored for convergence, this introduces additional computational cost. Since the primary goal of the algorithm is accurate angle estimation, a more efficient stopping criterion based on convergence of the angular deviations is adopted.

### 5.4. Computational Complexity

The computational complexity of the proposed VB-OG-SBL for AoA estimation in the RIS side on a per-iteration basis is given in Table 1. Adding the steps in the table leads to the overall computational complexity of $O\left(J\cdot L+J^{2}.T_{2}+J^{3}+N_{Obs}.T_{2}.J\right)$. In practice, the number of sources (J) is limited, and the array size is larger than the observation length $\left(T_{2}<L\right)$; therefore, the final approximate computational complexity is $O\left(T(J\cdot L+N_{Obs}.T_{2}.J)\right)$, where T is the number of iterations. Traditional SBL has a relatively large computational complexity due to full inversion of the dictionary matrix, but reducing the dictionary matrix to the number of multipaths greatly reduces the final complexity.

The SOMP algorithm, on the other hand, has a computational complexity of $O\left(J.T_{1}.G_{M}^{2}\right)$ for AoD estimation and $O\left(J.T_{2}.\Phi .G_{R}^{2}\right)$ for the AoA estimation, which can increase substantially with the size of the dictionary. The overall complexity of the VB-OG-SBL algorithm is less than the complexity that would be required if refinement was carried out using a high-resolution dictionary in traditional SOMP.

## 6. Channel and Location Estimation

To estimate the channel coefficients and the location of the MS, first, the estimated AoA and AoD angles must be paired in the correct order. Then, the ToA for each path also should be estimated before the localization algorithm.

### 6.1. Permutation-Based AoA–AoD Mismatch Mitigation and Channel Coefficient Estimation

In sparse channel estimation frameworks with double-sided angular sparsity, estimating the AoAs and AoDs independently may result in an angular mismatch, where the AoA and AoD are not in a matching order. Given that both estimation phases are carried out on the same path and that estimated coefficients for both estimation phases should be approximately the same, we propose a simple two-step AoA–AoD matching method.

In the first step, the channel coefficients are calculated based on MS-side AoD estimation observations. The order of the estimated AoAs is taken as a reference, i.e., the AoAs are fixed in the order returned by the angular estimation algorithm, while for the AoDs, all the J! permutations are considered. For each permutation of AoD and the reference order AoA, a corresponding set of path gain coefficients $h^{\left(p\right)}$ is estimated using the following expression, where (p) is the permutation order:

(41) $$h^{\left(p\right)}=\frac{Y_{MS}}{\Phi A_{Rx}A_{Tx}^{(p)H}}$$

The second step is the mean squared error (MSE) calculation step. To identify the correct AoA–AoD pairing, each candidate $h^{\left(p\right)}$ is evaluated against the AoD candidates using an MSE criterion. For the *p*th candidate of *h* and the *i*th candidate of AoD, a reconstruction error is computed for the RIS-side observations:

(42) $$E_{p,i}={\left\|Y_{R}-Q\cdot A_{Rx}\cdot diag(h^{\left(p\right)})\cdot {\left(A_{Tx}^{\left(i\right)}\right)}^{H}\right\|}_{F}^{2}$$

The optimal pairing is selected as the permutation index *p*,*i* that minimizes the reconstruction error:

(43) $$p,i=\underset{p,i}{\arg\min}E_{p,i}$$

The correct pairing also gives us the channel coefficient for the paths. The protocol is simple with low computational complexity. For example, in this study, the number of paths to be estimated is 3; therefore, there are only 6 permutations to go over, which makes 36 error calculations. Although in practical implementations the number of NLoS paths is expected to remain low, for channels with more than 5 multipath components, the computational complexity of the method can increase rapidly. To overcome this, the estimated AoAs and AoDs should be grouped based on their unit direction vectors. These unit vectors represent the spatial orientation of the rays relative to the RIS and MS positions. Using this directional information, the rays can be grouped into smaller subsets of three or four based on their geometric alignment. The permutation-based matching algorithm can then be applied within each group rather than over the full set of angles.

### 6.2. ToA Estimation

To estimate the ToA of each multipath component in the RIS-MS channel, the channel coefficients obtained over *N_s_* subcarriers in the previous step are concatenated into a vector for each individual multipath component, $h_{j}={\left[h_{j,1},\ldots ,h_{j,N_{s}}\right]}^{T}$. The resulting vectors are correlated with a dictionary matrix, where each column of the dictionary corresponds to a distinct time-delay value [18,57,58]. Although prior work proposes the use of the DFT matrix due to its orthogonal columns, the resolution limitations of the DFT restrict its practical applicability. Therefore, a high-resolution dictionary matrix is constructed, containing finely sampled candidate time-delay values. The ToA estimates are then obtained by identifying the columns with the maximum correlation value.

Next, channel coefficients are concatenated into a vector for each multipath, $h_{j}={\left[h_{j,1},\ldots ,h_{j,N_{s}}\right]}^{T}$, where $N_{s}$ is the number of measurements for each multipath. The resulting vectors are correlated with a dictionary matrix, where each column of the dictionary corresponds to a distinct time-delay value [18,57,58]. The ToA estimates are obtained by identifying the columns with the maximum correlation value:

(44) $$m_{j}=\underset{m}{\arg\max}(D^{H}h_{j})$$

The resolution and the accuracy of ToA estimation is constrained by the available signal bandwidth and the number of subcarriers; thus, increasing the bandwidth and the number of subcarriers can improve the achievable ToA accuracy.

### 6.3. Localization and Sensing

The 3D localization estimation framework deployed utilizes the geometric parameters that have been estimated by the estimation algorithms. Hence, the 3D coordinates of the BS and the RIS are known, and the location of the MS is estimated relative to the RIS location. Each propagation path consists of two segments, and the two-segment equation is given as follows:

(45) $$P_{M}=P_{R}+d_{j,1}u_{R,j}+d_{j,2}u_{M,j}$$

where $u_{R,j}$ and $u_{M,j}$ are the unit directional vectors for AoA at the RIS and AoD at the MS; $d_{j,1}$ and $d_{j,2}$ denote the two-segment structure of each path, where $d_{j,1}$ is the distance from the RIS to the *j*th SP and $d_{j,2}$ is the distance from the MS to the *j*th SP. The total distance of each path is known, $d_{j}=\tau_{j}.c$. The unit vector in the equation is

(46) $$u=\left(\begin{array}{c} \sin\theta \cos\phi \\ \sin\theta \sin\phi \\ \cos\phi \end{array}\right)$$

Using the available data, the MS location relative to the RIS position can be determined by optimizing the orthogonal distances between the potential intersection point and the multipath components [6,10,59]:

(47) $$P_{M}={\left(\sum_{j=1}^{J}w_{j}(I-{\bar{u}}_{j}{{\bar{u}}_{j}}^{T})\right)}^{-1}\sum_{j=1}^{J}w_{j}(I-{\bar{u}}_{j}{{\bar{u}}_{j}}^{T})\kappa_{j}$$

In the equation, $u_{j}=d_{j}(u_{R,j}-u_{M,j})$ is the path direction and ${\bar{u}}_{j}=u_{j}/\left\|u_{j}\right\|$ is the normalized unit direction vector; $\kappa_{j}=-d_{j}u_{M}$ is the directional vector from the MS, and $w_{j}$ is the weights representing the estimation quality of the path. Using the estimated MS location, the location points of the scatter points relative to the RIS position are calculated as follows:

(48) $$P_{SP}^{\left(j\right)}={\left(V_{R,j}+V_{M,j}\right)}^{-1}(V_{M,j}P_{M})$$

where $V_{R,j}=I-u_{R,j}u_{R,j}^{T}$ and $V_{M,j}=I-u_{M,j}u_{M,j}^{T}$.

The localization algorithm implemented in the study assumes a known orientation. Given the current available constraints, orientation estimation cannot be successfully performed. However, with added constraints such as known scatter point locations or other means, the orientation can be estimated. Moreover, most mobile devices are equipped with gyroscopes or other onboard sensors that can successfully track MS orientation; thus, the MS can inform the BS of its orientation by sending data packets in the data frames shown in Figure 2.

## 7. Results

This section presents simulation results in order to evaluate the performance of the proposed hybrid SOMP and VB-OG-SBL channel estimation framework. To benchmark the proposed method, simulations are also conducted using conventional SOMP and compressive MUSIC [60]. The standard SBL algorithm is excluded from the evaluation due to its high computational cost in large-scale RIS scenarios; for instance, the RIS dictionary in the study is of size $A_{R}\in {\mathbb{C}}^{144\times 8100}$. To provide a more comprehensive evaluation of the VB-OG-SBL algorithm, we also implement two Expectation Maximization (EM)-based OG-SBL variants as baselines: one that employs the same Newton-style angular refinement used in our proposed method and another that relies on a conventional grid-search refinement strategy [61].

In the simulations, the location of the RIS is set to $P_{R}={\left[0,0,10\right]}^{T}$ and the location of the MS is $P_{M}={\left[25+\sigma_{x},0+\sigma_{y},0\right]}^{T}$, where random numbers of standard deviation σ=1 are added to the x and y coordinates to avoid estimation bias. Scatterer positions are set to $P_{S}^{\left(1\right)}={\left[13,5,8\right]}^{T}$, $P_{S}^{\left(2\right)}={\left[7,-3,3\right]}^{T}$, and $P_{S}^{\left(3\right)}={\left[16,-9,5\right]}^{T}$. Hence, the MS location is measured with respect to RIS, and the BS position is not taken into account. The normal of RIS is aligned with the positive x-axis. The path gains are calculated according to 3GPP specifications for the UMi–Street Canyon profile, $PL(dB)=22.4+21.3\log_{10}\left(f_{c}\right)+35.3\log_{10}\left(d_{j}\right)-0.3(h_{U}-1.5)$, with $d_{j}$ indicating the total multipath distance and $h_{u}$ the MS height [62]. The OFDM parameters are B=200 MHz, N=200, and $f_{c}=28\;\mathrm{GHz}$.

The elements spacing for all the arrays is d=λ/2. The MS is modelled with realistic UPA sizes of K=4×4=16, K=5×5=25, and K=6×6=36. The RIS size is L=12×12=144, and to simulate various compression ratios, RIS observations are sampled at $T_{2}=\{15,20,30,48\}$, corresponding to sampling ratios of 10.4%,  13.8%, 20.8% and 33.3%.

The pilot sequences are transmitted over 10 OFDM subcarriers, which are evenly spaced through the entire bandwidth. These subcarriers are also used for ToA estimation, i.e., $N_{s}=10$. MMV matrices are constructed by taking observations from 3 BS antenna elements for each subcarrier, making the total observation size 30. Based on the compressed sensing bounds [47,48], the required number of observations is approximated as $N_{Obs}=cJlog\left(G_{M}^{2}/J\right)$; considering that both the AoA and AoD dictionaries span 90° for azimuth and elevation with angular resolution of 1°, the required MMV size for the successful estimation of AoA and AoD is 27 observations for c=1, which is consistent with the choice of 30 observations.

Estimation performance is evaluated in terms of NMSE for channel estimation and root-mean-squared error (RMSE) for angular and localization accuracy. Given H and P are the values to be estimated, $H_{Est}$ and $P_{Est}$ are the estimated values, and S is the number of number of samples, NMSE and RMSE are calculated as follows:

(49) $$NMSE=\frac{1}{S}\sum_{s=1}^{S}\frac{{\left\|\left.H_{Est}-H\right\|\right.}_{F}^{2}}{\left\|{\left.H\right\|}_{F}^{2}\right.}$$

(50) $$RMSE=\sqrt{\frac{1}{S}\sum_{s=1}^{S}{\left(P_{Est}-P\right)}^{2}}$$

The first set of simulations investigates the effectiveness of the hybrid angle estimation method. Coarse SOMP estimation as well as the baseline MUSIC algorithm have grid resolutions of 1°. For AoD estimation, the RIS-side compressive sensing matrix size is 25×144, while for AoA estimation, the MS UPA size was varied.

**Figure 3:**
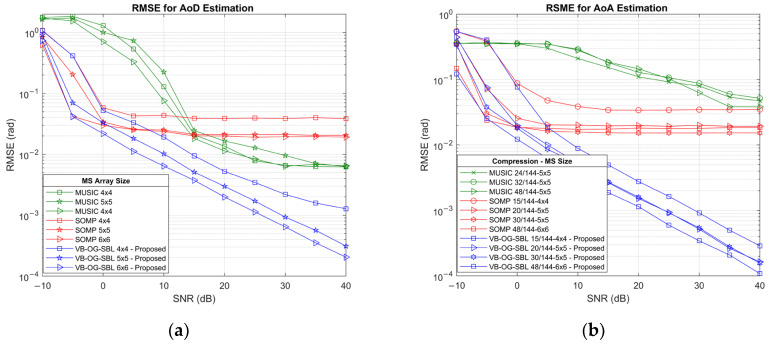
RMSE for (**a**) AoD estimation at the MS side, (**b**) AoA estimation at the RIS side.

The simulation results as a function of SNR for AoD and AoA estimations are displayed in Figure 3. Observing the results for the AoD estimation, it is clear that the proposed VB-OG-SBL algorithm performs as intended; the RMSE for coarse SOMP estimates has reduced from 0.038 rad to $1.3\times 10^{-3}\;\mathrm{rad}$ for 4×4 UPA and from 0.02 rad to $2.04\times 10^{-4}\;\mathrm{rad}$ for 6×6 UPA at 40 dB. The performance of the SOMP algorithm improves with array size, and it reaches its saturation point at about 5 dB SNR, reflecting its ability to recover support early but also showing its limited resolution at high SNR. The results for MUSIC show that the algorithm performs poorly below 10 dB SNR due to subspace instability and peak detection errors. However, its performance improves at higher SNRs, and it outperforms SOMP. The VB-OG-SBL refined results consistently achieve the lowest RMSE across all SNR levels and array sizes. Performance improves steadily with increasing SNR, and the improvement is more pronounced with larger arrays. Notably, the RMSE results for 5×5 and 6×6 arrays exhibit linear decay on a log scale, which suggests stable convergence and high-resolution capability. Refined results for the 4×4 array are also near-linear but with signs of saturation beyond 30 dB.

Compared with the AoD estimation, the AoA estimation results show very similar RMSE trends to the SOMP algorithm. Specifically, the SOMP RMSE for AoA estimation flattens at approximately 0.035 rad for a RIS compression ratio of 15/144 and at around 0.018 rad for higher compression ratios. The starting point of the flattening at low SNR is an indicator for the importance of the size of the compression matrix. The results for compressive MUSIC show that the algorithm is sensitive to RIS compression, with performance consistently lagging behind both SOMP and VB-OG-SBL across all SNR levels. This is attributed to subspace leakage and peak detection errors exacerbated by compression. The VB-OG-SBL refinement results for AoA estimation follow the same trend as in AoD estimation. While the RMSE for compression ratios of 30/144 and 48/144 are relatively linear beyond −5 dB, the results for compression of 20/144 stabilize at 0 dB, showing the negative effects of compression at low SNR. Moreover, the RMSE for the more aggressive compression at 15/144 show it stabilizes around 5 dB . The relatively linear decrease observed on the logarithmic scale confirms the stability of the algorithm. The results further demonstrate that VB-OG-SBL is robust under compressed sensing conditions and capable of exploiting spatial structure, even with an aggressive sampling rate of 10.4%.

To evaluate the computational feasibility of the proposed algorithm, runtime experiments were conducted using MATLAB R2023b on a PC with Intel Core i7-13700H CPU. The simulations were run for MS size of 5×5 and RIS compression of 20/144. The runtimes for various SNR values were averaged, and the final results are given in Table 2. The results confirm that SOMP is the fastest algorithm, as expected. Compressive MUSIC had the worst performance out of the tested algorithms, showing that not only its RMSE performance but also its computational cost under the 2D UPA model limits its practicality for the tested scenario.

The next sets of simulations are aimed at further observing the effectiveness of the proposed VB-OG-SBL algorithm. First, its performance is compared against a conventional EM-OG-SBL method with the same second-order Newton refinement and grid-based search. Starting with the computation time analyses, it can be seen that the proposed VB-OG-SBL algorithm outperforms both EM-OG-SBL methods. EM-OG-SBL with grid-based search is slow, as expected, because it searches through a refined grid looking for the best log likelihood estimate. Comparing VB-OG-SBL and EM-OG-SBL with the identical second-order Newton refinements, the VB-OG-SBL is overall 67% quicker per estimation and 75% quicker per iteration. The two algorithms converge at a similar iteration number. The computation time differences are primarily caused by the posterior update mechanisms. The EM framework performs full maximization of the log-likelihood, involving matrix inversions and explicit recomputation of the posterior covariance matrices. Conversely, the VB-OG-SBL method relies on variational inference, where posterior updates are expressed in terms of expectations and structured updates. Moreover, the two methods have different convergence criteria, but this does not seem to have a major impact. The EM framework converges based on the monotonic ascent of the log-likelihood function, while VB-OG-SBL uses a stopping condition based on the relative change in angle updates.

**Figure 4:**
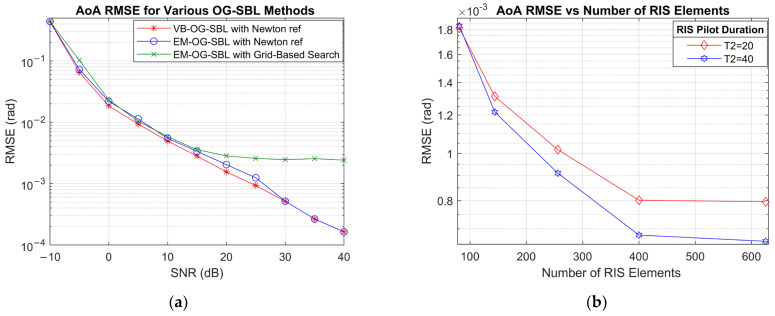
RMSE for (**a**) AoA estimation for various OG-SBL methods and (**b**) AoA estimation vs. number of RIS elements at SNR = 20 dB.

Figure 4a displays the RMSE results for AoA estimation as a function of SNR for the three OG-SBL methods. The results show that VB-OG-SBL has marginally better RMSE performance compared with EM-OG-SBL with Newton refinement, particularly in the low-to-mid SNR regime (0–20 dB). This performance gain could be attributed to the VB-OG-SBL maintaining and updating full posterior distributions, which leads to more stable convergence and stronger regularization, especially under low SNR or high compression. EM-OG-SBL, in contrast, uses a point-estimate-driven Expectation Maximization strategy that maximizes the marginal likelihood. Lastly, the EM-OG-SBL with grid-based search reaches a relatively flat RMSE trend beyond 15 dB. The flat performance is due to the limited angular resolution imposed by the search grid.

**Figure 5:**
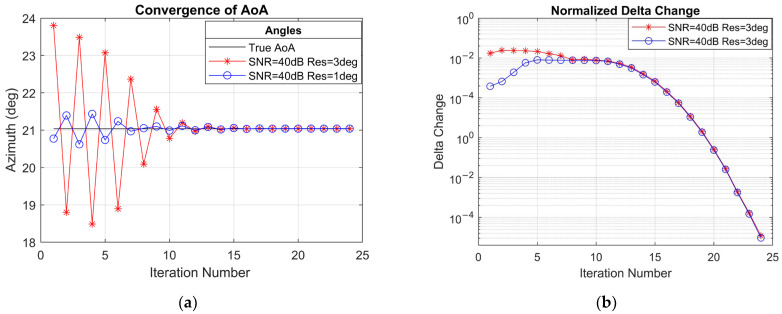
Convergence of VB-OG-SBL (**a**) shown as updated angles for resolution of 3 degrees and 1 degree coarse estimates and (**b**) shown as normalized angle update change in radians.

The convergence behaviour of the VB-OG-SBL method is displayed in Figure 5a,b, which depict the change in the estimated angular parameters and the corresponding reduction in the normalized angle change across iterations. The convergence criteria for the VB-OG-SBL algorithm are set to $norm(delta\_change)<10^{-5}$. Figure 5b clearly shows the normalized angle update reaching a point below $10^{-5}$ at iteration 24. Figure 5a further demonstrates how the angles are updated iteratively and approximate the true AoA values. It can be concluded from Figure 5a that AoA estimation reaches a reasonable value at the $10^{th}$ iteration, after which the subsequent updates serve as fine-tuning. This early convergence is an expected behaviour, as both VBEM-based SBL algorithms and second-order Newton updates promote early convergence.

Another important observation from Figure 5a is the ability of the VB-OG-SBL algorithm to correct coarse values from a wide range of initial values, provided that the initializations fall within a reasonable proximity to the true angle. With the current simulations, the update limit is set to 10 degrees. This highlights the algorithm’s robustness in correcting coarse values.

Figure 4b shows the effects of RIS size on the AoA RMSE performance; data was generated at 20 dB SNR and MS size of 5×5. The effects of RIS size were evaluated for training durations of 20 and 40 OFDM symbols. As expected, the estimation accuracy improves with larger RIS sizes due to the enhanced angular resolution enabled by the increased aperture. Notably, the RMSE decreases rapidly up to approximately 400 elements, beyond which the performance gain saturates. This result also demonstrates that in addition to the RIS size, the training duration, which is effectively the compression rate, is also important.

**Figure 6:**
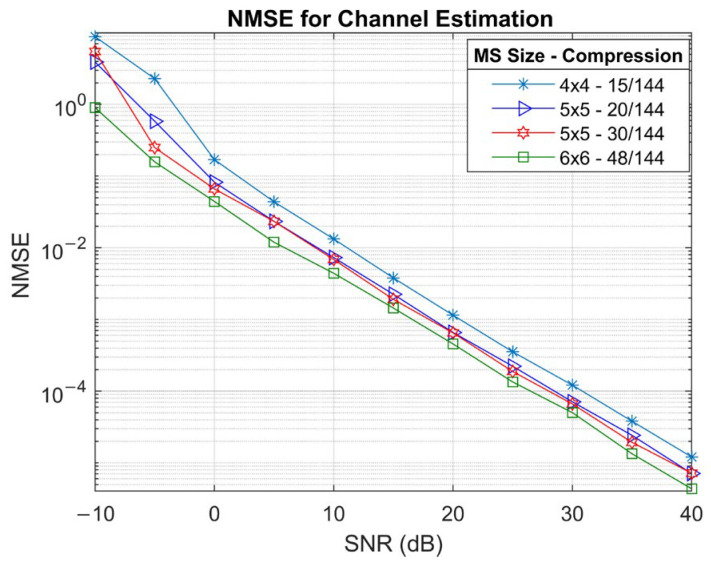
NMSE simulation results for various MS and RIS configurations.

Figure 6 illustrates the NMSE for RIS-MS channel estimation as a function of SNR. As expected, larger MS arrays provide improved estimation performance; specifically, the 6×6 MS UPA consistently achieves the lowest NMSE across all SNR levels, outperforming 4×4 UPA by approximately 5 dB. At SNR below 0 dB, NMSE differences between configurations are more pronounced, with the smallest array and most compressed setup performing the worst. Although there seems to be a marginal difference between the NMSE curves for 5×5 arrays with 20/144 and 30/144 compression ratios, beyond 0 dB, the two curves almost converge. This indicates that the algorithm is more robust to variations in sampling rate at higher SNR. In addition, NMSE improves with increasing SNR for all configurations, with no observable performance saturation even at high SNR.

**Figure 7:**
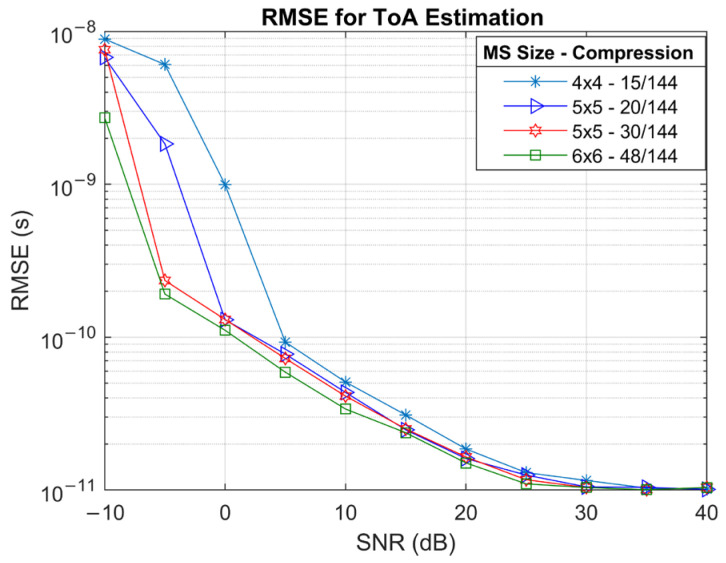
RMSE simulation results for ToA estimation for various MS and RIS configurations.

Figure 7 presents the ToA estimation accuracy in terms of RMSE across a range of SNR values. The results demonstrate a strong dependence on SNR, with all configurations showing steep improvements in ToA accuracy as SNR increases. The RMSE improves rapidly from $10^{-8}\;s$ to below $10^{-10}\;s$ as SNR increases from –10 dB  to 5 dB . This indicates that ToA estimation is primarily noise-limited and benefits substantially from higher signal quality. Comparing different configurations, there are 3 dB  to 5 dB differences between 4×4 and 6×6 UPA arrays at mid-SNR ranges, which was also observed in the channel estimation. At higher SNRs, the RMSE curves for all configurations converge, demonstrating that the algorithm reaches similar resolution limits when the signal is sufficiently strong. ToA estimations follow a similar trend to the channel estimation, as ToA estimation uses the estimated channel coefficients.

**Figure 8:**
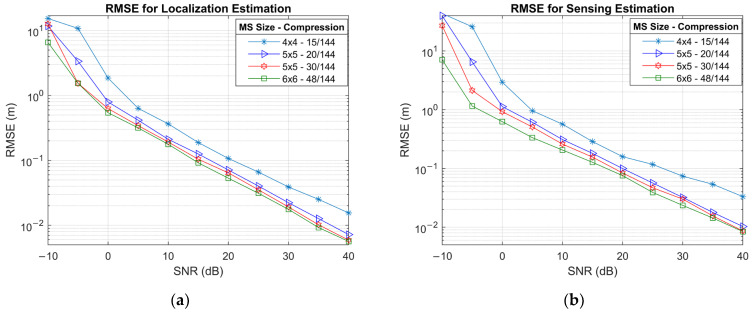
RMSE simulation results for (**a**) MS localization and (**b**) scatter point sensing.

**Figure 9:**
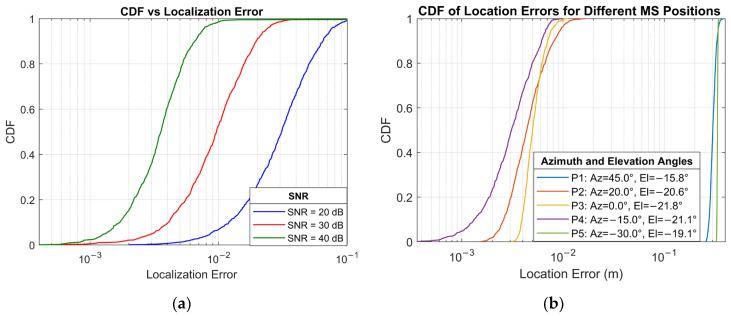
CDF for localization error (**a**) with changing SNR values and (**b**) with changing angles.

Figure 8 presents the RMSE for 3D localization and environmental sensing as a function of SNR for various configurations, while Figure 9a shows the same localization results as a cumulative distribution function (CDF). Since 3D localization is extremely sensitive to estimation errors in AoA, AoD, and ToA, the localization accuracy reflects the compounded effect of these parameters. For example, the lower performance of a 4×4 UPA array is a result of AoD estimation starting to saturate at higher SNR, as shown in Figure 3a. As expected, configurations with larger MS arrays and higher RIS sampling ratios lead to better localization accuracy. The 6×6 MS UPA with 48/144 compression outperforms the other cases, reaching sub-metre accuracy beyond −3 dB and centimetre-level RMSE at around 29 dB. Comparing 20/144 and 30/144 compression ratios for 5×5 MS UPA also confirms that a higher sampling ratio offers better localization performance. Both compression ratios for 5×5 MS UPA reach centimetre-level RMSE beyond 35 dB.

The results for scatter point sensing are presented in Figure 8b. Hence, environment sensing relies on MS localization, and the RMSE results mirror the plot for MS localization with only small differences in the RMSE values. At low SNR values, the sensing RMSE is marginally lower than 3D localization RMSE, likely due to the geometric proximity of the scatterers to the RIS, which intuitively reduces propagation error. However, as SNR increases and localization accuracy improves, the performance gap reverses slightly, and sensing becomes marginally less accurate than localization. This inversion arises from the compounded sensitivity of sensing to small localization errors; i.e., when localization accuracy reaches centimetre-level precision, even slight deviations are amplified in scatterer estimations.

The CDF data for the error distributions shown in Figure 9a,b was generated with MS size 5×5 and compression ratio of 30/144. Rather than the general trend of the localization estimation, the CDF plots in Figure 9a show the statistical distribution of localization error for the tested SNR values. The results show that at 40 dB SNR, 97% of the results are under 0.01 m, and at 30 dB, half the samples fall below 0.01 m. In contrast, at 20 dB, only 7% of the samples are below 0.01 m. In the tested conditions, close to 100% of the samples are below 1 m.

The CDF plot in Figure 9b was generated by moving the MS along the y-axis to different positions, thereby introducing different azimuth angles relative to the x-axis, which serves as the incidence plane for the RIS surface. In this configuration, the AoAs remain fixed, while the AoDs vary with respect to the fixed scatterers. This setup allows a systematic angular sweep to examine the limitations of the proposed channel estimation and localization framework. Overall, the results confirm that certain azimuth configurations perform better, as shown by the CDF curves for positions P2 (Az = 20.0°), P3 (Az = 0.0°), and P4 (Az = −15°). These positions produce distinct, well-separated paths towards the scatterers, enabling the localization algorithm to resolve multipath components accurately. In contrast, the P1 (Az = 45.0°) and P5 (Az = –30.0°) configurations demonstrate degraded performance, with the majority of the sample errors being around 40 cm. In these two settings, while the RIS-related AoA remains fixed, the change in AoD causes the departure paths towards the scatterers to become highly correlated. As a result, the localization algorithm faces increased difficulty in resolving the MS position due to insufficient angular diversity. From an algorithmic point of view, the coarse angles produced by the SOMP algorithm were beyond recoverable by the VB-OG-SBL algorithm.

While P1 (Az = 45.0°) and P5 (Az = –30.0°) scenarios represent valid edge cases, it must be emphasized that localization algorithms inherently rely on the angular separability of multipath components. In configurations where the AoD geometry consists of similar or collinear propagation paths, the underlying assumption of distinct paths no longer holds. From a practical perspective, these edge cases do not necessarily illustrate localization algorithm failure. The observed degradation in performance under these settings is consistent with theoretical expectations.

## 8. Discussion

This section discusses the practical implications of these findings, including deployment suggestions and the inherent limitations of the current study, which suggest avenues for future work.

### 8.1. Deployment Considerations

The proposed framework is designed with practical implementation in mind, offering a scalable and efficient solution for next-generation wireless systems.

Computational Feasibility: The proposed hybrid approach addresses computational complexity by using a low-complexity SOMP algorithm for coarse estimation. The more intensive VB-OG-SBL with Newton refinements is applied only to the coarse angles, significantly reducing the overall computational demand. The results have confirmed that the proposed VB-OG-SBL outperforms conventional EM-based OG-SBL algorithms by 67%, making it suitable for resource-constrained networks.

Training Overhead: Adapting VB-OG-SBL to compressive sensing SSRs in large RISs is one of the major contributions of this work. Localization with the MS array of size 4×4 and 15/144 compression is a demonstration of the edge scenario for both the MS UPA size and the RIS compression. This configuration shows that by using 16 OFDM symbols for AoD estimation and 15 symbols for AoA estimation, with a total of 31 symbols, it is possible to achieve 1.55 cm localization RMSE at 40 dB with the help of VB-OG-SBL. Furthermore, the framework used only 10 OFDM subcarriers, making the algorithm spectrally efficient.

Deployment in Dense Urban Environments: The algorithm is specifically designed for NLoS conditions that are common in dense urban settings. The algorithm was tested for various MS angles, and it was shown to be effective as long as there are distinct NLoS multipath components.

### 8.2. Practical Limitations and Future Research Directions

Several assumptions are made by the study that need to be highlighted, and future research areas regarding these assumptions should be made.

Known BS–RIS Channel and Orientation: The study assumes that the geometric parameters of the BS-RIS channel are known in advance. While this link is calibrated in fixed infrastructure, in some practical RIS deployments it would need to be estimated, as localization algorithms are very sensitive to imperfect orientation angles. This assumption requires equipping the RIS with orientation and location estimation sensors, which should periodically send information to the BS.

Hardware and System Imperfections: This study assumes an ideal system model. In practice, RIS elements typically provide only discrete phase shifts [14,63]. However, the proposed algorithm remains compatible with such quantized settings, provided that the resulting RIS patterns approximate the RIP. Other hardware impairments such as phase noise, RF non-linearities, and synchronization errors may degrade estimation accuracy. A thorough investigation of the algorithm’s robustness under these conditions is essential for real-world deployment.

User Mobility: The algorithm assumes static or quasi-static MS during training. In high-mobility scenarios, rapid channel variation invalidates this assumption. For the framework to be applicable to many 6G use cases such as vehicle localization, it must be extended to high-mobility scenarios. This would require incorporating channel-tracking mechanisms to adapt to a dynamically changing environment.

Orientation Estimation: The localization algorithm assumes the orientation of the MS is known. While most mobile devices are equipped with sensors like gyroscopes that can provide this information, a fully autonomous system would benefit from estimating orientation directly from the wireless signals. Future work could explore adding constraints such as known scatterer locations or multiple RIS deployments to enable joint position and orientation estimation.

Multiple RIS Deployments: The proposed algorithm is inherently adaptable to multi-RIS configurations. In synchronous deployments, signals reflected from multiple RISs can be superimposed at the receiver, allowing joint estimation of multiple AoAs and AoDs. However, this requires the development of an additional association algorithm to correctly match angular components to their corresponding RISs. Alternatively, by allocating distinct subsets of OFDM subcarriers to each RIS, the angles can be estimated and combined for enhanced angular diversity and localization accuracy.

Exploring Learning-Based Localization: An emerging trend is the use of deep learning models for RIS-assisted localization. These data-driven methods can complement geometric models by capturing environment-dependent features, enabling robust estimation even under ambiguous or non-ideal propagation conditions [64]. Hybrid frameworks that fuse statistical learning with physical modelling offer a promising path forward. However, deep learning methods still rely on training datasets often generated via traditional model-based estimation. As such, the current framework remains highly relevant, serving both as a standalone solution and as a data-generation backbone for learning-based approaches.

## 9. Conclusions

The objective of the study was to solve the problem of uplink channel and localization estimation in the RIS-MS channel of RIS-assisted MIMO-OFDM systems in dense urban environments where only NLoS components are available and the communicating nodes are modelled as UPAs. To achieve its objectives, the study first defined the RIS-MS channel as a double-sparsity structure and proposed a hybrid estimation framework. The proposed hybrid estimation framework combines SOMP for coarse angular support detection with a VBEM-based off-grid SBL algorithm for refinement, where angular deviations are calculated using Newton’s method. The multiple observations were obtained from OFDM subcarriers.

Simulation results show that the proposed hybrid method with VB-OG-SBL refinement outperforms the conventional EM-based OG-SBL in AoA estimation and computation times. The proposed algorithm was shown to achieve high-resolution angular estimation in the $10^{-4}\;rad$ range; high localization accuracy, with 97% of the results within 0.01 m; and channel estimation error of 0.0046% at 40 dB SNR. In addition, the algorithm maintains estimation performance even under strong compression of 10.4%, outperforming conventional subspace methods, which degrade under similar conditions. It can therefore be concluded that the design choices were appropriate and the algorithm performs effectively in RIS-assisted systems.

Overall, the proposed hybrid VB-OG-SBL method offers a scalable, high-accuracy solution for uplink sensing and localization in RIS-assisted networks. It addresses key limitations of traditional sparse recovery techniques and enables robust geometric inference in compressed sensing regimes, paving the way for deployable sensing-enabled 6G systems.

## Figures and Tables

**Table 1:** Computational complexity of the proposed off-grid SBL per iteration.

Step	Computational Complexity
Steering vector updates	O(J·L)
E-step	$O\left(J^{2}.T_{2}+J^{3}\right)$ $+O\left(N_{Obs}.J.T_{2}\right)$
M-step	$O\left(N_{Obs}.J\right)$ $+O\left(N_{Obs}.T_{2}.J\right)$
Angle refinement	$O\left(N_{Obs}.T_{2}.J\right)$

**Table 2:** Execution time and iteration comparison for methods.

Method	Avg. Time (ms)	Avg. Iterations	Time/Iter. (ms)
SOMP	10.08	-	-
Compressive MUSIC	303.72	-	-
VB-OG-SBL	16.18	27.6	0.59
EM-OG-SBL with Newton	26.97	26.1	1.03
EM-OG-SBL with grid search	99.98	10.8	9.26

## Data Availability

The source code used in this study is currently being prepared for public release and will be uploaded to a GitHub repository following publication. In the meantime, the code and simulation data are available upon request from the authors.

## Abbreviations

The following abbreviations are used in this manuscript:

RIS	Reconfigurable Intelligent Surface
OFDM	Orthogonal frequency division multiplexing
MIMO	Multiple-input multiple-output
MS	Mobile station
UPA	Uniform planar array
AoA	Angle of Arrival
AoD	Angle of Departure
SOMP	Simultaneous Orthogonal Matching Pursuit
VBEM	Variational Bayesian Expectation Maximization
SBL	Sparse Bayesian Learning
OG-SBL	Off-Grid Sparse Bayesian Learning
NMSE	Normalized mean square error
RMSE	Root-mean-square error
SNR	Signal-to-noise ratio
LoS	Line-of-sight
NLoS	Non-line-of-sight
MMV	Multiple measurement vector
ToA	Time of Arrival
MUSIC	Multiple signal classification
ESPRIT	Estimation of Signal Parameters via Rotational Invariance Techniques
VLoS	Virtual line-of-sight
SP	Scatter point
LS	Least squares
CoSaMP	Compressive Sampling Matching Pursuit
MP	Matching Pursuit
OMP	Orthogonal Matching Pursuit
SSR	Sparse signal recovery
ELBO	Evidence lower bound
SISO	Single-input single-output
mmWave	Millimetre-wave
CDF	Cumulative distribution function
EM	Expectation Maximization
